# A Dual-Mode Electrochemical Aptasensor for Ochratoxin A Detection in Red Wine Based on a Target-Induced Displacement Strategy

**DOI:** 10.3390/foods15213940

**Published:** 2026-11-03

**Authors:** Xueying Yu, Zhaoxian Hou, Yi Zhi, Shuo Zhang, Yuping Wen, Zhijun Guo

**Affiliations:** 1 Key Laboratory of Natural Medicines of the Changbai Mountain, Ministry of Education, Yanbian University, Yanji 133002, China; 2 College of Agriculture, Yanbian University, Yanji 133002, China

**Keywords:** ochratoxin A, dual-mode aptasensor, target-induced displacement, electrochemical impedance spectroscopy, differential pulse voltammetry, red wine

## Abstract

Ochratoxin A (OTA) is a mycotoxin of concern in wine and food safety, highlighting the need for reliable detection in complex food matrices. In this study, a dual-mode electrochemical aptasensor was developed for OTA determination in red wine based on a target-induced displacement strategy. OTA binding to its aptamer induces dissociation of the aptamer from complementary DNA (cDNA) pre-immobilized on the electrode surface, thereby altering the interfacial environment and enabling detection by electrochemical impedance spectroscopy (EIS) and differential pulse voltammetry (DPV). The sensor showed a semi-logarithmic calibration relationship over the experimentally tested OTA concentration range of 0.05–10 ng/mL. The estimated EIS limit of detection (LOD) was 0.018 ng/mL; however, this value was below the lowest tested concentration and was not experimentally validated. For DPV, 0.05 ng/mL represents the lower limit of the tested range rather than an established LOD. In spiked red wine, recoveries ranged from 94.6% to 103.9%, with relative standard deviations below 10%. The dual-mode strategy provides two readouts of the same target-induced interfacial change and offers potential for preliminary OTA screening in red wine with simplified sample pretreatment.

## 1. Introduction

Ochratoxin A (OTA), a highly toxic mycotoxin widely distributed in agricultural and processed foods [1,2], is classified as a possible human carcinogen (Group 2B) by the International Agency for Research on Cancer (IARC) [3]. It is important to note that wine contamination poses a serious food safety issue. The European Food Safety Authority (EFSA) has included wine among the food categories that may lead to dietary exposure to OTA; other categories include cereals and cereal products, beer, grape juice, coffee, cocoa products, and pork [4]. To mitigate this pervasive risk, strict regulatory thresholds (e.g., a maximum allowable limit of 2.0 ng/mL) have been globally established [5,6,7]. Consequently, developing rapid, sensitive, and on-site detection methodologies for OTA in complex wine matrices is of paramount importance for food safety monitoring [8].

Traditional methods for detecting OTA, such as high-performance liquid chromatography (HPLC) [9,10,11], liquid chromatography-tandem mass spectrometry (LC-MS/MS) [12,13,14], enzyme-linked immunosorbent assay (ELISA) [15], and thin-layer chromatography (TLC) [16], offer advantages including high accuracy, strong regulatory acceptance, and reliable quantitative performance. However, when applied to wine analysis, these methods exhibit significant limitations because they rely on laboratory settings, require expensive large-scale instruments, and depend on trained technical personnel [17]. Moreover, wine samples may require pretreatment, including centrifugation, filtration, dilution, or extraction and cleanup procedures such as liquid–liquid extraction (LLE), solid-phase extraction (SPE), and immunoaffinity-column cleanup. These procedures may increase analysis time and cost, limiting the suitability of conventional methods for rapid on-site screening [18,19]. Conversely, electrochemical detection offers a straightforward and potentially cost-effective alternative for preliminary screening. However, conventional electrochemical biosensors frequently rely on “signal-off” or competitive formats. For instance, Karczmarczyk et al. [20] established a notable precedent by developing an electrochemical sensor based on a competitive immunoassay for OTA detection in wine. Although their work demonstrated the feasibility of electrochemical analysis in wine matrices, the reliance on exogenous labels and the inherent limitations of the competitive format resulted in restricted sensitivity, highlighting the urgent need for more sensitive methodologies.

To address these sensitivity challenges, studies on electrochemical aptasensors have explored target-induced displacement (TID) as well as other signal-generation and interface-design strategies, including dual catalytic hairpin assembly and tetrahedral DNA nanostructures [21,22]. The TID approach traces back to the foundational work of Li et al. [23], who leveraged target-triggered strand release to reconfigure the electrode interface for faster charge transfer. Building directly on this switching concept, Zhang et al. [24] migrated the TID mechanism to mycotoxin assays, demonstrating that the physical shedding of a pre-hybridized DNA element effectively blunts matrix interference. Subsequent developments shifted toward interface and substrate engineering; for instance, Ma et al. [25] optimized hybridization kinetics to maximize switching efficiency, while Jampasa et al. [26] expanded the system’s adaptability by integrating TID onto modified paper-based sensing electrodes. Despite these advancements, reported TID tools remain restricted to a solitary readout mode. When facing complex matrices like red wine, co-existing components—particularly ethanol and phenolic compounds—can alter the electrochemical behavior of the target or foul the electrode surface. Without an additional electrochemical readout, single-mode systems provide fewer opportunities for internal comparison when interpreting responses in complex matrices.

However, conventional signal-on displacement-based aptasensors heavily rely on tedious covalent labeling of redox tags, which often compromises the native binding affinity of aptamers and increases fabrication complexity. Meanwhile, interpretation based on a single electrochemical readout may be more difficult when matrix-related signal variations occur in complex samples. To address these limitations, a dual-mode electrochemical aptasensor based on a target-induced displacement mechanism was developed. This displacement process is proposed to reduce the density of negatively charged aptamers at the interface, thereby diminishing both electrostatic repulsion and steric hindrance toward [Fe(CN)_6_]^3−/4−^ redox probes. Consequently, this interfacial gating mechanism triggers a synchronized response, manifested as a simultaneous increase in DPV peak current and a corresponding decrease in charge-transfer resistance (R_ct_) [27,28]. In this work, ΔR_ct_ and ΔI_p_ showed a strong positive correlation, indicating that the two readouts changed consistently with OTA concentration. The platform operates as a “signal-on” system, where the target-induced release of the aptamer facilitates the access of redox probes to the electrode surface. The proposed platform is label-free because no redox label is attached to the aptamer or cDNA. However, it is not reagentless, as electrochemical measurements require an externally added [Fe(CN)_6_]^3−/4−^ redox probe in solution. By combining electrochemical impedance spectroscopy (EIS) and differential pulse voltammetry (DPV), two complementary electrochemical responses can be obtained from the same sensing system. A good correlation was observed between the two signals, allowing the DPV and EIS results to be compared with each other and providing additional support for the detection results. Overall, the proposed platform provides a simple proof-of-concept strategy for OTA detection and preliminary screening in wine samples.

## 2. Materials and Methods

### 2.1. Materials and Chemicals

The OTA aptamer (5′-GAT CGG GTG TGG GTG GCG TAA AGG GAG CAT CGG ACA-3′) was originally selected by Cruz-Aguado and Penner [29] and was subsequently used in an electrochemical OTA aptasensor [30]. The thiolated complementary cDNA sequence (5′-SH-TGT CCG ATG CTC CCT TTA CGC CAC CCA CAC CCG ATC-3′) [31] served as the electrode-immobilized capture strand. Both oligonucleotides were synthesized and purified by Sangon Biotech Co., Ltd. (Shanghai, China). The cDNA was modified with a thiol group (-SH) at the 5′ terminus via a hexyl linker to facilitate immobilization onto the gold electrode surface through stable Au–S bonding. Prior to use, the cDNA was activated with Tris(2-carboxyethyl) phosphine hydrochloride (TCEP) to reduce disulfide bonds. Lyophilized DNA strands were reconstituted in ultrapure water to prepare stock solutions and stored at −20 °C.

TCEP (98%, CAS No.: 51805-45-9), 6-mercapto-1-hexanol (MCH, 98%, CAS No.: 1633-78-9), potassium ferricyanide (K_3_[Fe(CN)_6_], 99.5%, CAS No.: 13746-66-2), and potassium ferrocyanide trihydrate (K_4_[Fe(CN)_6_]·3H_2_O, 99.0%, CAS No.: 14459-95-1) were all purchased from Shanghai Macklin Biochemical Technology Co., Ltd., Shanghai, China. Potassium nitrate (KNO_3_, 99%, CAS No.: 7757-79-1) was obtained from Sinopharm Chemical Reagent Co., Ltd., Shanghai, China. A 1× phosphate-buffered saline solution (PBS, pH 7.4, without calcium and magnesium ions, sterile-filtered) was purchased from Beijing Labgic Technology Co., Ltd., Beijing, China. Standard substances, including OTA, ochratoxin B (OTB), ochratoxin C (OTC), aflatoxin B1 (AFB_1_), aflatoxin M1 (AFM_1_), and N-Acetyl-L-phenylalanine (NAP), were obtained from Sigma-Aldrich (St. Louis, MO, USA). OTA (98%, Sigma-Aldrich) was dissolved in methanol to prepare a stock solution (1 mg/mL) and stored at −20 °C in the dark. Working solutions with different concentrations were prepared by serial dilution with 1× PBS (pH 7.4) before use. The red wine samples were all purchased from local supermarkets. All other reagents were of analytical grade and used as received without further purification. Ultrapure water (resistivity of 18.2 MΩ·cm at 25 °C) was supplied by a Milli-Q ultrapure water system (Millipore Corporation, Bedford, MA, USA).

### 2.2. Apparatus and Instruments

All electrochemical measurements were conducted at room temperature using a CHI 660E electrochemical workstation (CH Instruments Shanghai Co., Ltd., Shanghai, China). Electrochemical impedance spectroscopy (EIS) and differential pulse voltammetry (DPV) were employed for surface characterization and quantitative detection. A standard three-electrode system was employed, consisting of a gold working electrode (AuE, 3 mm in diameter), a Ag/AgCl reference electrode filled with saturated KCl solution, and a platinum wire counter electrode, all of which were supplied by Shanghai Yue Ci Electronic Technology Co., Ltd. (Shanghai, China). An LC-HN-25BS electrothermal incubator (Lichen Scientific Instrument (Zhejiang) Co., Ltd., Shaoxing, China) was used for incubation. Ultrasonic cleaning was performed with a KQ-400DE ultrasonic cleaner (Kunshan Ultrasonic Instrument Co., Ltd., Kunshan, China). A high-purity nitrogen stream was used for drying steps.

### 2.3. Preparation of the Aptasensor

Prior to modifying the working electrode surface, it was sequentially polished with a suede polishing cloth using 1.0 μm, 0.3 μm, and 0.05 μm alumina polishing powder for 1–2 min each until a mirror-like finish was achieved. The electrode was then ultrasonically cleaned with anhydrous ethanol and ultrapure water for 2 min each, followed by drying with nitrogen blowing [32]. The electrode was then immersed in a 0.5 M H_2_SO_4_ solution and activated by cyclic voltammetry (CV). The potential was scanned from −0.2 to 1.0 V at a scan rate of 50 mV/s for twenty cycles until a stable CV curve was obtained [33,34].

The cDNA was activated by treating it with 4 mM TCEP at room temperature for 80 min to reduce its disulfide bonds. The molar concentration ratio of cDNA to TCEP was 1:2000 (2.0 μM:4 mM). A 10 μL aliquot of the activated cDNA solution (2.0 μM) was immobilized onto the surface of a clean gold electrode and incubated at 37 °C for 120 min, allowing the cDNA to facilitate firm attachment to the gold electrode via Au-S bonds. The electrode was then rinsed with 1× PBS, dried, and incubated with 1 mM MCH for 60 min. This strategic application of MCH prior to hybridization serves a dual purpose: it blocks remaining active sites to minimize non-specific adsorption and displaces loosely adsorbed nucleobases, promoting an upright orientation of the cDNA strands. This “standing up” conformation is expected to minimize steric hindrance and potentially improve the spatial accessibility of cDNA for subsequent hybridization. Following this, 10 μL of a diluted 4 μM aptamer solution (diluted from the stock solution using phosphate buffer, PB, pH 7.4) was applied to the surface of the blocked gold electrode and incubated at 37 °C for 80 min, enabling it to hybridize with the cDNA immobilized on the electrode surface and form a double strand. Notably, no thermal denaturation or annealing pretreatment (e.g., 95 °C heating) was applied to the aptamer solution prior to hybridization. This direct assembly approach was strategically preferred to prevent competitive intramolecular self-folding of the free aptamer, thereby maximizing its accessibility and intermolecular hybridization efficiency with the surface-confined cDNA. All incubation steps were performed in a sealed, humidified environment. Finally, the electrode, after undergoing these multiple modification steps, was incubated with OTA solutions of varying concentrations for subsequent data measurement.

### 2.4. Electrochemical Parameter Settings

Prior to electrochemical impedance spectroscopy (EIS) measurements, the open circuit potential (OCP) was recorded to ensure system stability. Based on this voltage, the frequency range was set from 0.01 Hz to 100 kHz, with an AC amplitude of 5 mV and a quiet time of 10 s.

For the differential pulse voltammetry (DPV) measurements, the following parameters were applied: a potential range from 0 to 0.5 V, a pulse amplitude of 50 mV, a pulse width of 50 ms, a step potential of 4 mV, and a quiet time of 10 s.

All experiments were conducted in an electrolyte containing 5 mM [Fe(CN)_6_]^3−/4−^ with 0.4 M KNO_3_. Electrochemical data were acquired using a CHI 660E electrochemical workstation. After each modification and measurement step, the electrode surface was rinsed 2–3 times with 1× PBS and dried under a nitrogen stream before proceeding to the next measurement. For experiments conducted in triplicate, three independently prepared aptasensors were tested under identical conditions (*n* = 3). Results are expressed as mean ± standard deviation (SD), with error bars representing variability among independently prepared aptasensors.

### 2.5. Optimization Procedures for Aptasensor Fabrication and Detection

To achieve optimal sensing performance, three key parameters governing the sensor fabrication were systematically investigated. First, by monitoring the trend in impedance changes on the electrode after immobilizing cDNA at different concentrations, the optimal cDNA immobilization concentration was determined. Here, the hybridization signal increment (ΔR_ct_, defined as the difference in R_ct_ before and after aptamer hybridization) was used as the key evaluation index for optimizing cDNA immobilization time.

Second, analysis of impedance values obtained from different cDNA immobilization times led to the identification of the optimal cDNA immobilization duration. Subsequently, to achieve the highest hybridization efficiency and verify the role of the blocking agent, impedance measurements were performed on electrodes with and without MCH blocking conditions. This allowed for a comparison of the effect of different cDNA immobilization times on the subsequent aptamer hybridization signal. Finally, to achieve rapid and sufficient detection, the incubation time for the aptamer with the target OTA was optimized.

### 2.6. Detection of OTA in Spiked Red Wine Samples

Red wine samples were purchased from a local supermarket (produced in Northeast China). Following the confirmation of OTA absence by high-performance liquid chromatography (HPLC), a 5 mL sample was collected. This sample was then diluted with 10 mL of 1× PBS buffer, shaken for 2 min, and subsequently filtered through a 0.22 μm aqueous filter membrane. Before OTA spiking, the resulting solution represented a threefold dilution of the original wine matrix. The filtrate was collected, and different concentrations of OTA standard solution were added. The OTA content was then evaluated using the aptamer sensor constructed in this study. The gold electrode modified with cDNA-MCH-Apt was immersed in the prepared red wine sample solution and incubated at 37 °C for 60 min. Following rinsing and drying, the electrode was immersed in a 5 mM [Fe(CN)_6_]^3−/4−^ solution containing 0.4 M KNO_3_ for measurement.

## 3. Results and Discussion

### 3.1. Principle of the Constructed Dual-Mode Electrochemical Aptamer Sensor

**Scheme 1:**
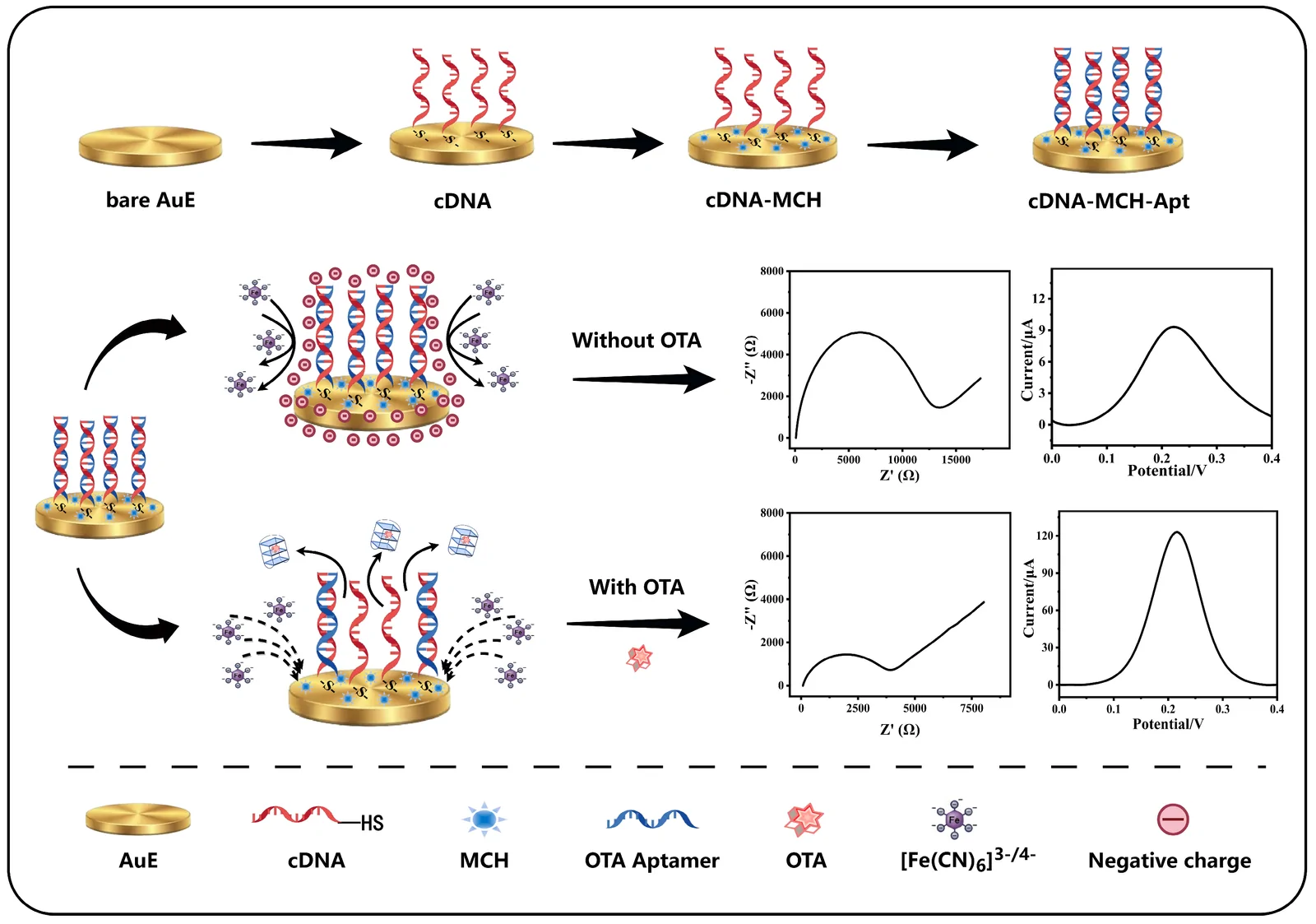
Schematic illustration of the electrochemical aptasensor fabrication and detection mechanism.

The working principle of the designed electrochemical aptasensor is illustrated in Scheme 1. The sensing platform operates via a target-induced displacement mechanism. Upon OTA binding to the aptamer, the surface-confined aptamercDNA duplex may be destabilized or partially disrupted [35], potentially altering the interfacial structure and charge distribution and thereby modulating the electron transfer efficiency of the electrochemical probe. The proposed displacement process may alter the interfacial structure and charge distribution, thereby modulating the electron transfer behavior of the electrochemical probe. Consequently, the sensitive detection of OTA is achieved by recording the corresponding changes in charge-transfer resistance (R_ct_) and Faradaic current.

Label-free electrochemical sensing strategies have been described in terms of blocking or opening access to the electrode surface [36]. In the present aptasensor, OTA-induced aptamer displacement increases the accessibility of the electrode surface to the redox probe. Specifically, the thiolated cDNA is immobilized onto the gold electrode surface via stable Au–S bonding. MCH is subsequently employed to block remaining active sites, aiming to minimize non-specific adsorption and improve the signal-to-noise ratio. The aptamer then hybridizes with the cDNA to form a rigid double-stranded DNA (dsDNA) structure. In this blank state (without OTA), the long-chain dsDNA structure carries a high density of negative charges from its phosphate backbone, which acts as an efficient electrostatic barrier and generates strong steric hindrance. Consequently, it is difficult for the anionic [Fe (CN)_6_]^3−/4−^ redox probes in the solution to reach the electrode surface, leading to a maximum R_ct_ value.

Upon the introduction of OTA, binding of OTA to the aptamer may destabilize or partially disrupt the aptamer–cDNA duplex [35]. These interfacial changes may reduce the effective electrostatic and steric barriers to the redox probe, facilitating electron transfer. This interpretation is consistent with the observed decrease in R_ct_ and increase in the DPV peak current, although these electrochemical changes do not establish complete aptamer dissociation. The changes in the two electrochemical signals were well correlated with OTA concentration, enabling dual-mode detection through the target-induced displacement mechanism.

A point worth clarifying is the order of MCH blocking relative to aptamer hybridization. In this work, MCH blocking was performed prior to aptamer hybridization. This order was chosen to minimize nonspecific adsorption of the aptamer onto bare gold sites, which could otherwise lead to high background signals. This sequence not only ensures that the aptamer binding occurs exclusively on the cDNA-modified regions but also promotes a better interfacial environment for hybridization by promoting an upright orientation of cDNA strands, which is expected to mitigate steric hindrance and favor plausible spatial accessibility for subsequent hybridization. A similar strategy has been reported in previous studies [37].

### 3.2. Characterization of the Constructed Electrochemical Aptamer Sensor

To comprehensively characterize the sensor fabrication process, electrochemical impedance spectroscopy (EIS) and differential pulse voltammetry (DPV) were employed to systematically monitor the changes on the electrode surface. The Nyquist plots were fitted with the equivalent circuit model using ZView (v2.0) software. To ensure the rigor of the EIS analysis, the fitting quality was comprehensively evaluated via multiple indicators: the chi-squared (χ^2^) values were consistently on the order of 10^−3^ to 10^−4^ with negligible residuals, and the relative errors for the core fitted parameters (solution resistance, constant phase element (CPE), and charge-transfer resistance (R_ct_)) were well below 5%, demonstrating a highly reliable mathematical agreement between the experimental and simulated spectra. All presented DPV curves were baseline-corrected and fitted using the Multi-Peak Fit function in Origin software to accurately extract the peak currents (I_p_).

**Figure 1:**
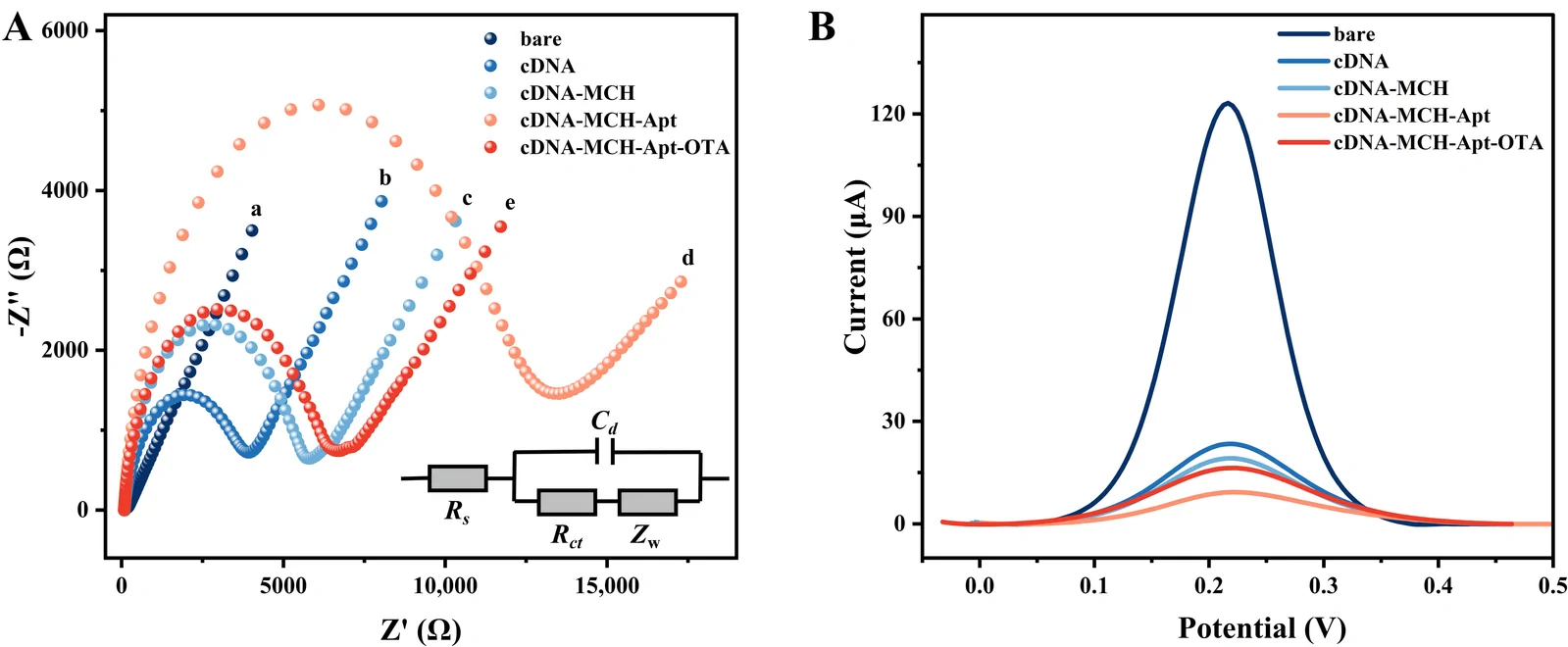
(**A**) Electrochemical impedance spectroscopy (EIS) Nyquist plots of electrodes at different modification stages and (**B**) differential pulse voltammetry (DPV) responses: (a) bare electrode, (b) complementary DNA (cDNA)-modified electrode, (c) cDNA-MCH-modified electrode, (d) cDNA-MCH-aptamer (Apt)-modified electrode, and (e) cDNA-MCH-Apt-ochratoxin A (OTA)-treated electrode. After cDNA immobilization, MCH blocking, and aptamer hybridization, the semicircle diameter (R_ct_) increases, indicating the progressive formation of an interfacial barrier due to enhanced steric hindrance and electrostatic repulsion. Subsequent incubation with OTA decreases R_ct_ and restores the DPV peak current, consistent with the proposed aptamer displacement and partial disruption of the interfacial barrier.

As shown in Figure 1A, the EIS curve of the bare gold electrode exhibited a straight line with a large slope (curve a), and the semicircle in the high-frequency region was nearly absent, indicating an extremely low charge transfer resistance (R_ct_) value. After immobilizing cDNA on the gold electrode, a distinct semicircle appeared in the curve (curve b) due to the electrostatic repulsion between the negatively charged phosphate backbone of the cDNA and the anionic [Fe(CN)_6_]^3−/4−^ probe, leading to a significant increase in R_ct_. When MCH was used to block the nonspecific binding sites not occupied by cDNA (curve c), a further gradual increase in R_ct_ was observed. Subsequently, the OTA aptamer was incubated on the gold electrode to hybridize with cDNA and form double-stranded DNA (dsDNA). Owing to the formation of the rigid double-stranded DNA (dsDNA) structure that held a high density of negative charges and formed a dense barrier, the R_ct_ value reached its maximum (curve d). Upon introduction of OTA, the observed decrease in R_ct_ was consistent with the proposed target-induced displacement of the aptamer from the electrode surface. This response may reflect partial dissociation of the aptamer from cDNA and a weakening of the interfacial blocking effect on electron transfer. This left the remaining cDNA to shift from rigid dsDNA into highly flexible single-stranded DNA (ssDNA) that tended to collapse. This structural change, along with the loss of surface negative charges from the removed aptamer, effectively opened up the interfacial pathways for the redox probe, resulting in a marked decrease in R_ct_ (curve e). These consistent electrochemical changes support the successful construction of the sensor and are consistent with the proposed OTA-induced interfacial displacement process.

As shown in Figure 1B, the bare electrode exhibited the highest peak current (curve a). With the immobilization of complementary DNA, blocking with MCH, and the formation of double-stranded DNA, the peak current decreased sequentially (curves b–d), reaching its lowest value after aptamer hybridization (curve d). Upon the introduction of OTA, a distinct recovery of the peak current was observed (curve e). Based on the changes in peak current, the decrease and subsequent recovery of the current are consistent with the weakening and removal of the interfacial barrier to electron transfer of the probe. The consistent responses obtained from the two electrochemical readouts further support the proposed interfacial displacement mechanism. To understand this dual-mode sensing mechanism better, the individual effects of surface charge density and layer compactness on the electron transfer of [Fe (CN)_6_]^3^^−^^/4−^ were further distinguished. When the aptamer hybridizes with cDNA to form dsDNA on the electrode surface, it blocks the redox probe through both electrostatic repulsion and steric hindrance. On the one hand, the negative phosphate backbone of dsDNA repels the anionic probe. On the other hand, the rigid, vertical dsDNA structure forms a tightly packed layer that acts as a physical barrier. When OTA is introduced, the aptamer is displaced and released into the solution. This process not only reduces the negative charge density on the surface but also changes the remaining rigid structure into a more flexible single-stranded cDNA, making the bio-interface looser. As a result, the diffusion pathways open up, leading to the simultaneous increase in DPV peak current and decrease in charge-transfer resistance (R_ct_). Although direct surface characterization using SEM, AFM, or XPS was not performed, the proposed interfacial displacement mechanism was indirectly supported by comparative CV and peak-to-peak potential separation (ΔE_p_) measurements (Figure S3). Together, these results support the proposed target-induced rearrangement at the electrode interface.

Together, the EIS and DPV results indicate a signal-on response to OTA, reflected by a decrease in R_ct_ and an increase in I_p_. These changes are complementary electrochemical manifestations of the same interfacial process: OTA-induced aptamer displacement reduces electrostatic repulsion and steric hindrance, facilitating access of the [Fe(CN)_6_]^3−/4−^ redox probe to the electrode surface and promoting electron transfer.

### 3.3. Optimization of Experimental Conditions

To evaluate the dual-mode response, the critical parameters determining the interfacial assembly density and target-induced displacement kinetics were systematically optimized. While electrochemical impedance spectroscopy (EIS) was employed as the primary diagnostic tool to track the primary optimization trends, the corresponding DPV responses were also examined for consistency. As illustrated in Figure S2 (Supporting Information), the response profiles captured via DPV peak currents exhibit trends similar to those observed in the EIS mode. Specifically, the voltammetric response optimizes at a cDNA concentration of 2.0 μM (Figure S2A), a cDNA assembly time of 120 min (Figure S2B), and an OTA displacement incubation interval of 60 min (Figure S2C). This consistency is further supported by the correlation shown in Figure S1, where the analytical signal increments of the two modes (ΔI_p_ and ΔR_ct_) demonstrate a strong linear correlation (R^2^ = 0.99361). Since both modes are based on the same interfacial displacement process at the same electrode bio-interface, the optimal conditions established via EIS support the consistency of the DPV response under the optimized conditions, providing additional support for the dual-mode sensing strategy.

#### 3.3.1. cDNA Immobilization Concentration

**Figure 2:**
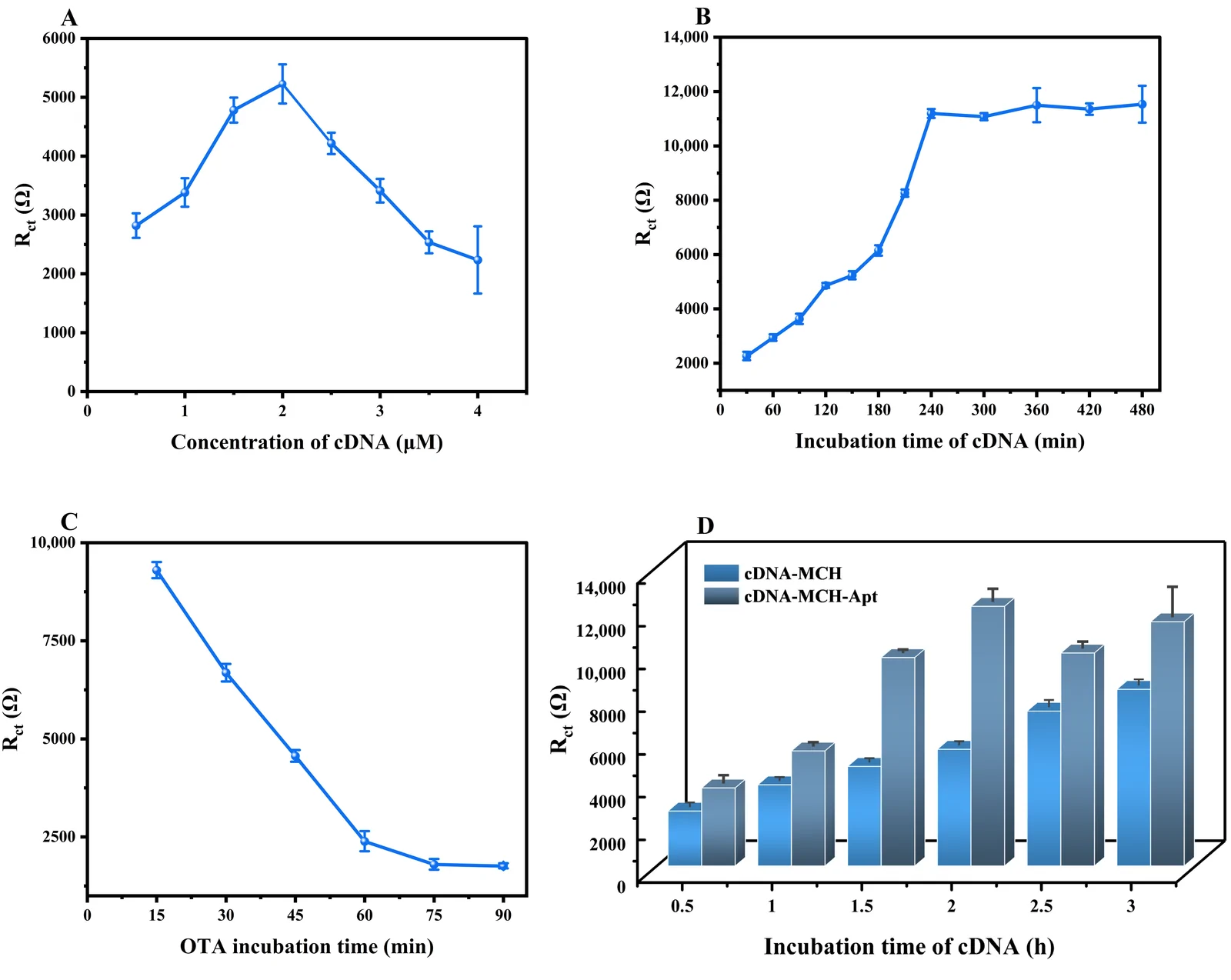
Optimization of key preparation parameters for the aptasensor based on EIS. (**A**) Effect of cDNA immobilization concentration (0.5 μM to 4.0 μM) on charge transfer resistance (R_ct_). (**B**) Effect of cDNA immobilization time (30 min to 480 min) on R_ct_. (**C**) Effect of OTA incubation time (15 min to 90 min) on the impedance response of fully assembled sensors. (**D**) Optimization of cDNA incubation time based on hybridization signal increment. All EIS measurements were conducted in a solution containing 5 mM [Fe(CN)_6_]^3−/4−^ and 0.4 M KNO_3_. Error bars represent the standard deviation of three parallel independently prepared aptasensors (*n* = 3).

The surface coverage of cDNA is a critical determinant of aptamer hybridization efficiency and the subsequent target-induced displacement. As illustrated in Figure 2A, when the cDNA concentration was increased from 0.5 μM to 2.0 μM, the interfacial charge transfer resistance (R_ct_) gradually rose from 2778 Ω to a peak value of 5227 Ω, indicating a progressive self-assembly of cDNA on the gold surface. However, further increasing the concentration to 4.0 μM induced a distinct decline in R_ct_ (down to 2236 Ω). This phenomenon demonstrates that an excessively dense DNA probe layer is likely to induce severe steric hindrance and electrostatic repulsion, which potentially limits the spatial accessibility for subsequent aptamer hybridization. Consequently, 2.0 μM was selected as the optimal immobilization concentration to ensure an optimized microenvironment for interfacial recognition.

#### 3.3.2. cDNA Immobilization Time

As shown in Figure 2B, the absolute R_ct_ value exhibited a continuous increase from 30 min to 240 min, reaching 11195 Ω, followed by a slight decrease at extended durations, which could be plausibly attributed to unfavorable intermolecular orientations within an overly crowded assembly. To strike a balance between rapid assaying and sensor sensitivity, the assembly kinetics were further evaluated using the hybridization signal increment (ΔR_ct_ = R_ct, after_ − R_ct, before_). As depicted in Figure 2D, although the absolute R_ct_ was highest at 240 min, the ΔR_ct_ value reached its maximum of 6694 Ω at 120 min. This confirms that the hybridization efficiency between the aptamer and cDNA is kinetically optimized at 120 min, whereas an overly dense cDNA monolayer paradoxically hinders mass transfer and subsequent duplex formation. Therefore, 120 min was selected as the optimal cDNA incubation time to achieve superior sensitivity within a shortened fabrication cycle.

#### 3.3.3. OTA Incubation Time

The kinetics of the target-induced displacement reaction were monitored over a time range of 15 min to 90 min. As shown in Figure 2C, the R_ct_ value gradually decreased as the incubation time extended, consistent with the progressive target-induced displacement process upon OTA binding. The impedance response reached a steady plateau after 60 min, indicating that the electrochemical response had reached a stable state at the sensing interface. Considering both the analytical signal magnitude and operational efficiency, 60 min was established as the optimal incubation time for OTA detection.

### 3.4. Analytical Performance and Sensitivity of the Dual-Mode Aptasensor

#### 3.4.1. Sensitivity and Quantitative Analysis

To evaluate the sensitivity and reliability of the constructed aptamer sensor for OTA detection, the sensor was subjected to a specific binding reaction with OTA standard solutions at varying concentrations (0.05, 0.06, 0.08, 0.1, 0.5, 1.0, 5.0, and 10.0 ng/mL) under the optimal conditions determined in Section 2.5. These conditions included a cDNA immobilization concentration of 2.0 μM, an immobilization time of 120 min, MCH blocking for 60 min, and an OTA incubation time of 60 min. Detection verification was performed using electrochemical dual-mode analysis, specifically EIS and DPV.

**Figure 3:**
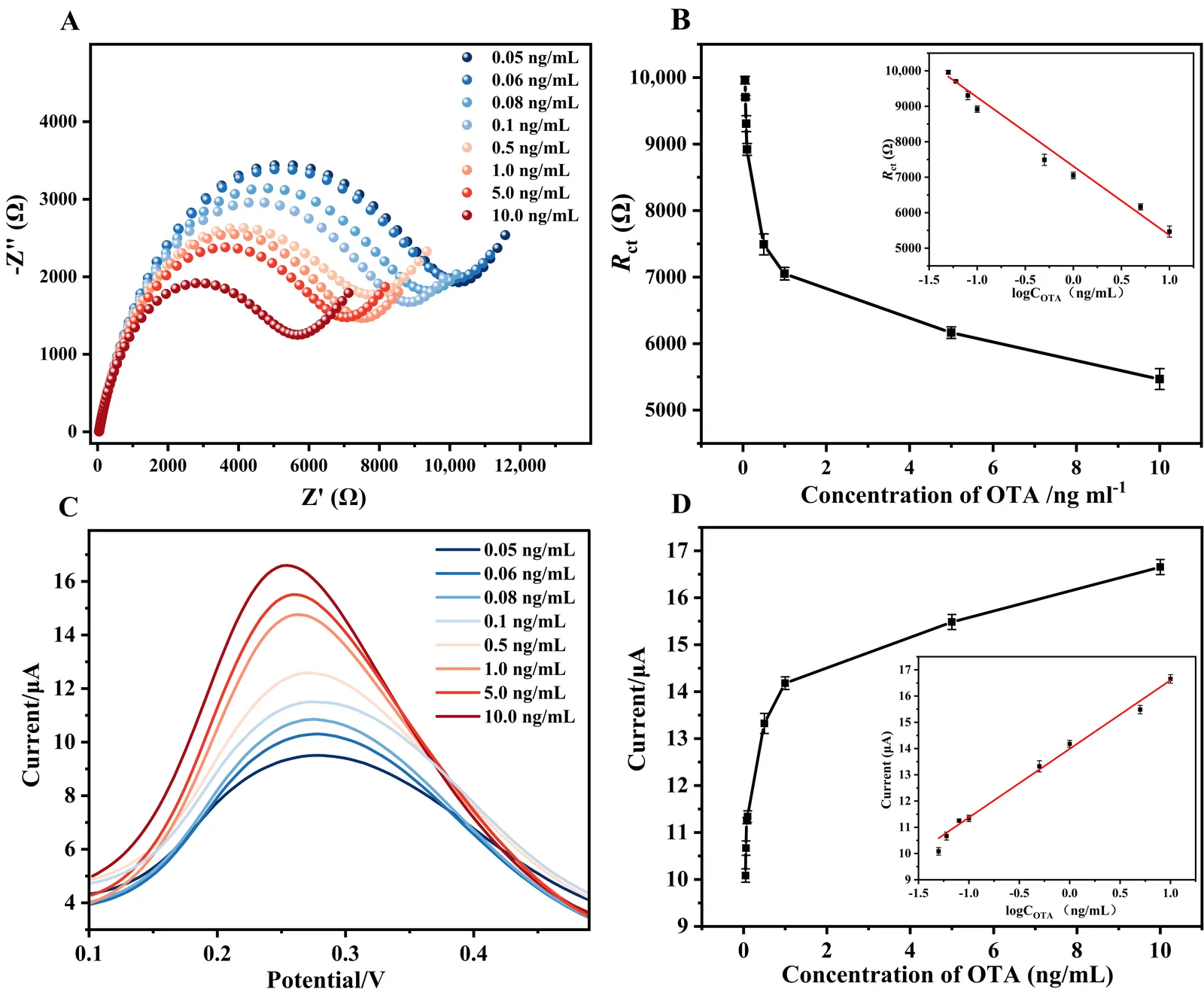
Performance analysis of the dual-mode aptasensor for OTA detection. (**A**) EIS responses and (**C**) DPV responses to different OTA concentrations (0.05–10 ng/mL). Corresponding calibration plots for (**B**) EIS and (**D**) DPV. Both the decrease in R_ct_ and the increase in peak current with rising OTA concentration reflect the same target-induced displacement process, which gradually disrupts the interfacial blocking layer. Error bars indicate the standard deviation obtained from three independently prepared aptasensors (*n* = 3).

As shown in Figure 3A, the charge transfer resistance (R_ct_) exhibited a gradual decrease as the OTA concentration increased from 0.05 to 10.0 ng/mL. This trend is consistent with the proposed target-induced displacement mechanism, which is expected to disrupt the dsDNA barrier at the electrode surface, with the extent of the electrochemical response increasing at higher target concentrations. Following linear fitting, the linear regression equation was obtained (Figure 3B) and is given by Equation (1):

(1) *y* = (7309.21 ± 107.31) − (1944.23 ± 93.53)*x*

where *x* = log *C*_OTA_, with *C*_OTA_ expressed in ng/mL. The linear correlation coefficient (*R*^2^) was 0.9863, indicating a linear relationship between the EIS response and the logarithm of OTA concentration over the experimentally tested range of 0.05–10 ng/mL. The estimated LOD was calculated using a 3*σ* signal-threshold criterion (Equation (2)), and the corresponding OTA concentration was determined using Equation (3):

(2) $$y_{\mathrm{th}}=\left\{\begin{array}{c} {\bar{y}}_{\mathrm{blank}}-3\sigma ,\;\mathrm{EIS}\\ {\bar{y}}_{\mathrm{blank}}+3\sigma ,\;\mathrm{DPV} \end{array}\right.$$

(3) $$C_{\mathrm{LOD}}=10^{(y_{\mathrm{th}}-a)/b}$$

where *σ* is the standard deviation of the blank R_ct_ responses for EIS, obtained from three parallel, independently prepared aptasensors. The EIS signal threshold was defined as the mean blank R_ct_ minus 3*σ*. Because the EIS calibration equation was expressed as *y* = *a* + *b* × log_10_(*C*), where C is the OTA concentration in ng/mL, the corresponding OTA concentration was calculated by substituting the signal threshold into the EIS calibration equation. Based on this criterion, the estimated LOD in the EIS mode was 0.018 ng/mL. Because this value is below the lowest experimentally tested concentration (0.05 ng/mL), it should be regarded as an extrapolated statistical estimate rather than an experimentally validated detection limit.

As shown in Figure 3C, the DPV results showed a trend consistent with the EIS results, supporting consistency between the two electrochemical readouts and the proposed interfacial process. With increasing OTA concentration, the faradaic peak current of the [Fe(CN)_6_]^3−/4−^ probe at the electrode surface showed a significant increasing trend. This indicates that the electrode interface blocking layer was weakened after target-induced displacement strategy, thereby enhancing the electron transfer efficiency of the probe. Following linear fitting, the linear regression equation (Figure 3D) is given by Equation (4):

(4) *y* = (13.99 ± 0.12) + (2.62 ± 0.12)*x*

where x is defined as log *C*_OTA_, with *C*_OTA_ expressed in ng/mL. The linear correlation coefficient (*R*^2^) was 0.9880, indicating a linear relationship between the DPV response and the logarithm of OTA concentration over the experimentally tested range of 0.05–10 ng/mL. For DPV, 0.05 ng/mL is the lowest experimentally tested concentration and is therefore reported as the lower limit of the tested range rather than as an established LOD. The estimated limits of quantification (LOQs), based on a 10*σ* signal-threshold criterion, were 0.0228 ng/mL for EIS and 0.1097 ng/mL for DPV. The estimated EIS LOQ was below the lowest experimentally tested concentration of 0.05 ng/mL, whereas the estimated DPV LOQ fell within the investigated calibration range of 0.05–10 ng/mL. However, both values represent statistical estimates rather than experimentally validated quantification limits. The estimation method is provided in Text S4 of the Supporting Information. The previously reported dissociation constant (K_D_) of the OTA aptamer was 0.2 μM under the aptamer selection conditions [29,38]. Although K_D_ reflects the binding affinity between the aptamer and OTA, the estimated LOD of the developed sensor also depends on the calibration slope and blank-response variability.

The decrease in R_ct_ and the increase in DPV peak current are complementary electrochemical responses, both indicating enhanced electron transfer of the [Fe(CN)_6_]^3−/4−^ probe at the electrode interface. The target-induced displacement strategy of OTA disrupts the dsDNA barrier, reducing interfacial steric hindrance and electrostatic repulsion. Consequently, the EIS R_ct_ decreases and the DPV faradaic current increases. The parallel trends in the two modes originate from the same mechanism and support the recognition event. Furthermore, the consistency between the DPV and EIS responses was quantitatively evaluated by correlation analysis. As illustrated in Figure S1, ΔI_p_ and ΔR_ct_ showed a strong positive linear relationship (ΔI_p_ = 0.00141 × ΔR_ct_ + 3.30336, *r* = 0.9968, *R*^2^ = 0.99361), indicating that the two electrochemical signals responded consistently to the same target-induced interfacial change. Since both signal variations arise from the same target-induced displacement process at the same bio-interface, the observed correlation supports the consistency of the dual-mode responses.

As summarized in Table 1, the proposed dual-mode aptasensor exhibited an estimated LOD of 0.018 ng/mL for EIS, while 0.05 ng/mL was the lowest experimentally tested concentration for DPV. Unlike conventional high-sensitivity electrochemical assays that rely on single-mode readouts or complex labeling procedures for signal amplification, our platform enables OTA quantification without covalently attached redox labels. Direct comparison of the two responses provides an additional consistency check without requiring additional instrumentation.

Compared with previously reported single-mode electrochemical OTA aptasensors [39,40,41], the present dual-mode aptasensor provides two electrochemical readouts for comparison within the same sensing system. Although some signal-amplification strategies, such as the DNA walker-driven system [42], achieve lower detection limits, the proposed platform combines label-free detection with dual-mode electrochemical analysis. In contrast, the electrochemical immunoassay reported by Karczmarczyk et al. [20] showed a higher detection limit of 15 ng/mL, reflecting the limitations associated with competitive assay formats. The present dual-mode aptasensor therefore combines an estimated EIS detection limit with complementary electrochemical readouts without requiring additional instrumentation. In complex red wine matrices, the agreement between the EIS and DPV responses provides additional information for interpreting the analytical results. Ma et al. [21] reported a low LOD using dual-CHA amplification, but their protocol included 60 min of aptamer–tDNA hybridization followed by a 120-min reaction. Xu et al. [22] also reported a low LOD using a tetrahedral DNA scaffold (TDN)-based interface, which required additional electrode-preparation steps. These examples illustrate the trade-off between analytical sensitivity and assay complexity. However, direct comparison of total analysis time remains difficult because complete sample-to-result times were not consistently reported.

Although the complete sensor preparation and assay require several hours, the method avoids the pre-enrichment and multi-step cleanup procedures commonly required for conventional HPLC analysis. The EIS and DPV responses can be compared to assess their consistency, providing additional information when interpreting the results. However, measurements in this study were performed using a conventional three-electrode system and a benchtop CHI 660E electrochemical workstation. Further integration with a portable electrochemical device is needed for practical on-site testing. Future work will focus on optimizing the interface assembly process and reducing incubation times to shorten the overall analysis time.

#### 3.4.2. Specificity Assessment of the Aptasensor

Given the consistent response trends and the agreement between the two electrochemical modes established in the previous sections, EIS was employed as the representative technique to evaluate the selectivity of the aptasensor. To evaluate the specific recognition performance of the constructed aptamer sensor, blank PBS buffer (1× PBS, pH 7.4) was selected as the blank control, while OTA served as the target analyte. Common mycotoxins and irrelevant small molecules found in food were chosen as interferents. OTB, a structural analog of OTA, AFB1, and AFM1 are mycotoxins that frequently coexist in wine. NAP, a common amino acid derivative in food, was also included. This diverse panel of interferents, particularly the structural analog OTB, was used to evaluate the molecular recognition specificity of the sensor. OTA and each individual interferent were tested at 5 ng/mL, and measurements were performed using three parallel, independently prepared aptasensors under identical experimental conditions (*n* = 3). The total concentration of the four interferents in the mixture was 20 ng/mL; OTA was not included.

**Figure 4:**
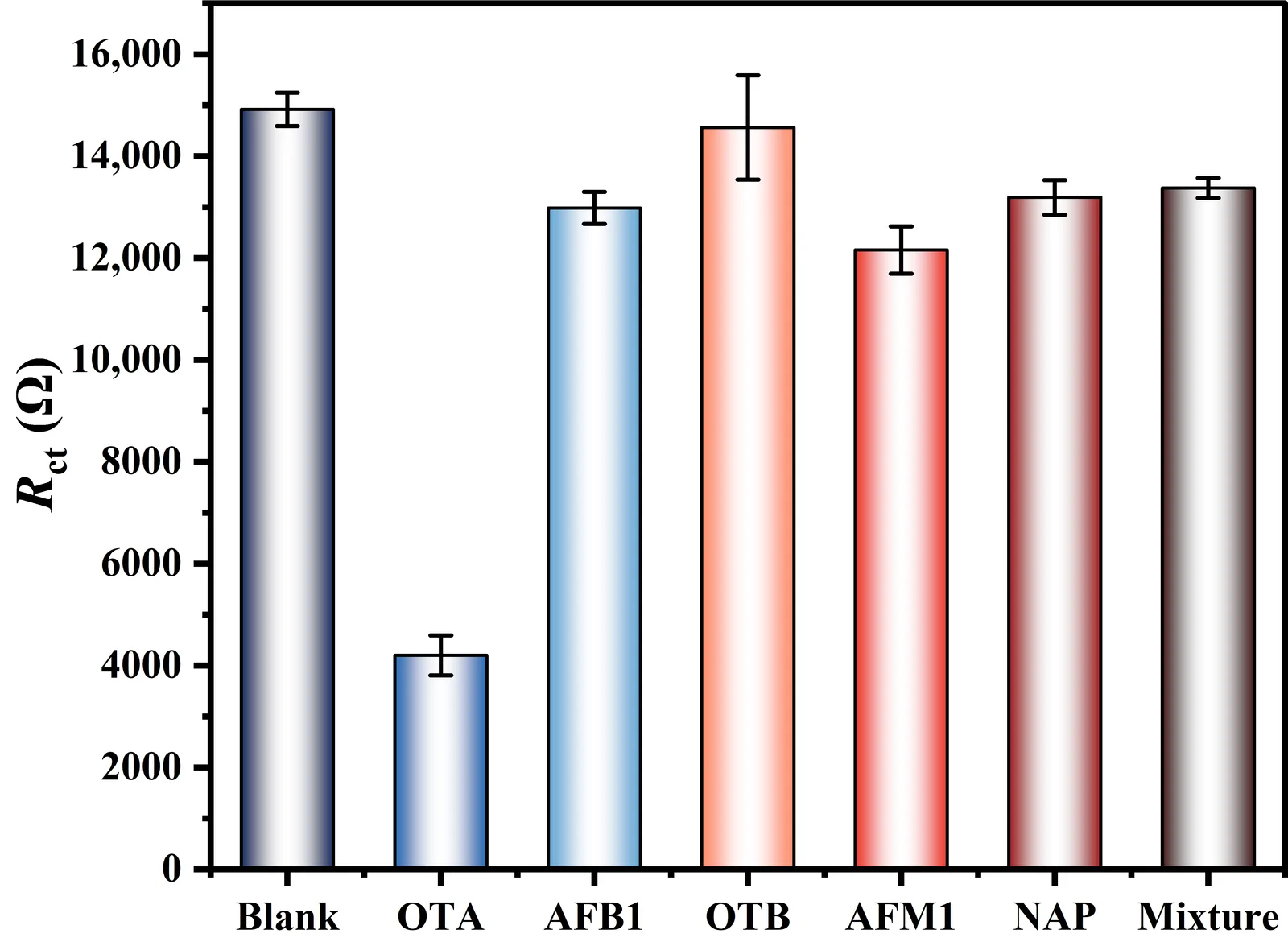
Specificity of the aptasensor for OTA detection. The sensor was incubated with 5 ng/mL OTA, OTB, AFB1, AFM1, NAP, Mixture and PBS buffer (blank). All measurements were conducted in 5 mM [Fe(CN)_6_]^3^^−^^/4−^ containing 0.4 M KNO_3_. Error bars represent the standard deviation obtained from three parallel, independently prepared aptasensors (*n* = 3). The interferent mixture contained OTB, AFB1, AFM1, and NAP at 5 ng/mL each (total interferent concentration, 20 ng/mL) and did not contain OTA.

As shown in Figure 4, the electrode impedance value for the blank PBS solution was similar to that of the electrode modified with the cDNA-MCH-Apt complex. The impedance response of the mixture was similar to that of the blank control and individual interferents, while a prominent impedance decrease was observed in the presence of OTA. These results indicate that the tested interferents, either individually or in combination, did not produce a pronounced OTA-like impedance response under the tested conditions. OTB was tested only at 5 ng/mL; therefore, potential cross-reactivity at higher concentrations, such as 50 ng/mL, cannot be excluded. However, a scrambled-DNA control was not included in this study, and further sequence-based validation is needed to provide additional evidence for aptamer-specific recognition.

#### 3.4.3. Analysis of Spiked Red Wine Samples

To assess the applicability of the constructed sensor to a diluted red wine matrix, blank red wine samples confirmed to be free of OTA by HPLC were selected as the matrix. It is worth noting that red wine possesses a highly complex matrix rich in ethanol and phenolic compounds (e.g., tannins and anthocyanins) [43]. These components can potentially compromise biosensor performance; for instance, ethanol may influence the stability of aptamer folding, while polyphenols are prone to non-specific adsorption on the electrode surface or may exhibit intrinsic electrochemical activity that overlaps with the redox probe signal. The red wine samples were diluted threefold with PBS (pH 7.4) before electrochemical detection to reduce the concentrations of matrix components. However, no ethanol-specific interference test was performed; therefore, the effect of residual ethanol on aptamer folding and cDNA–aptamer hybridization remains unverified. Dilution may also alter the solvent composition and ionic strength of the medium, potentially affecting the thermodynamic activity of OTA and its binding equilibrium with the aptamer.

Subsequently, spiked recovery experiments were conducted by adding standard OTA solutions at concentrations of 0.1 ng/mL, 1.0 ng/mL, and 5.0 ng/mL to the diluted blank samples. The analytical accuracy was evaluated using the recovery rate (%), calculated as follows: Recovery rate (%) = (Measured OTA concentration/Added OTA concentration) × 100%. At OTA spiking concentrations of 0.1, 1.0, and 5.0 ng/mL, the recovery rates were 95.3%, 103.9%, and 94.6%, respectively, with corresponding RSDs of 4.2%, 6.3%, and 9.7% (Table 2). These results demonstrate acceptable accuracy and precision under the tested red wine dilution and spiking conditions, supporting the applicability of the sensor for preliminary OTA screening in red wine. These recovery results were obtained using OTA-free red wine samples spiked with known OTA concentrations and therefore should be distinguished from validation using naturally contaminated red wine samples, which would provide a stronger assessment of the method’s performance in authentic samples.

In addition to the satisfactory recovery performance, the dual-mode strategy provides an additional consistency check compared with conventional single-mode methods. As shown in Figure S1, the strong positive linear correlation between ΔI_p_ and ΔR_ct_ (r = 0.9968, R^2^ = 0.99361) indicates that the two analytical signals changed consistently with the same target-induced interfacial response. When analyzing complex matrices such as red wine, comparison of the two electrochemical responses can provide additional information for assessing the consistency of the readouts.

## 4. Conclusions

In this work, a label-free, dual-mode electrochemical aptasensor based on a target-induced displacement strategy was successfully developed for the quantification of OTA in red wine. By integrating EIS and DPV on the same sensing interface, the platform provides two electrochemical responses that can be compared for consistency. The good agreement between the two readouts is consistent with the proposed target-induced interfacial response and provides additional information for result interpretation. This label-free configuration operates without the need for expensive chemical labeling of the aptamer sequence, thereby streamlining the assay preparation and minimizing potential impacts on target-binding affinity.

Under optimized experimental conditions, the constructed aptasensor demonstrated a semi-logarithmic calibration relationship over the experimentally tested OTA concentration range of 0.05 to 10.0 ng/mL. The estimated LOD for EIS was 0.018 ng/mL; however, this value was below the lowest experimentally tested concentration and should be regarded as an extrapolated statistical estimate rather than an experimentally validated detection limit. The estimated LOQs, based on the 10*σ* criterion, were 0.0228 ng/mL for EIS and 0.1097 ng/mL for DPV; these values represent statistical estimates rather than experimentally validated quantification limits. For DPV, 0.05 ng/mL represents the lower limit of the experimentally tested range rather than an established LOD. When applied to complex red wine matrices, the sensor exhibited acceptable analytical accuracy and precision, yielding recoveries of 94.6–103.9% with RSD values below 10% under the tested spiking conditions. Furthermore, specificity assessment showed that the tested interferents, individually or in combination, did not produce a pronounced OTA-like response under the tested conditions.

Overall, the proposed dual-mode aptasensor provides a proof-of-concept approach for preliminary OTA screening in red wine. Designed as a disposable platform, it does not require an electrode regeneration step after measurement. However, the measurements were performed using a conventional three-electrode configuration and a benchtop CHI 660E electrochemical workstation. Further integration with portable electrochemical devices will be needed for practical on-site applications. Storage stability at 4 °C and operational stability were not systematically evaluated in this study; therefore, further investigation is needed before practical application. Nevertheless, the present results demonstrate the feasibility of using dual electrochemical readouts for consistency checks.

## Figures and Tables

**Table 1:** Comparison of key performance parameters for OTA detection assays.

Signal Mode	Label-Free	Reported Reaction/Incubation Time	Calibration Range (ng/mL)	LOD (ng/mL)	Sample Matrix	References
Amp	No	50 min	15–100	15	Red wine	[20]
ACV	Yes	180 min	1 × 10^−3^–1 × 10^3^	9.2 × 10^−5^	Grains Red wine	[21]
DPV	Yes	40 min	0.001–8	6.8 × 10^−4^	Wheat flour	[22]
DPV	No	50 min	0.005–100	3.1 × 10^−4^	Red wine	[39]
SWV	No	180 min	0.001–500	5.64 × 10^−4^	Grains	[40]
DPV	No	30 min	0.002–2	8.1 × 10^−4^	Coffee	[41]
DPV	Yes	110 min	1 × 10^−4^–100	6.9 × 10^−5^	Red wine	[42]
EIS & DPV	Yes	60 min	0.05–10.0	0.018 (EIS, estimated)	Red wine	This work

Note: Reported reaction time refers to the reaction or incubation durations specified in the original studies. The included steps may differ among methods. Sample pretreatment, sensor fabrication, and electrochemical measurement are excluded. Because complete sample-to-result times were not consistently reported, the listed durations should not be interpreted as directly comparable total analysis times. Abbreviations: LOD, limit of detection; EIS, electrochemical impedance spectroscopy; DPV, differential pulse voltammetry.

**Table 2:** Recovery and precision of OTA in spiked red wine samples.

Sample	Added (ng/mL)	Mean Measured OTA Concentration (ng/mL)	95% CI (ng/mL)	Recovery (%)	RSD (%) (*n* = 3)	Relative Error (%)
A	0.1	0.0953	0.0854–0.1052	95.3	4.2	−4.7
B	1.0	1.039	0.876–1.202	103.9	6.3	3.9
C	5.0	4.73	3.59–5.87	94.6	9.7	−5.4

Note: The reported recovery and RSD values were obtained from three parallel, independently prepared aptasensors (*n* = 3). The 95% confidence intervals (CIs) were estimated from the reported mean concentrations and RSD values using Student’s t-distribution (*n* = 3, df = 2).

## Data Availability

The data presented in this study are available on request from the corresponding authors.

## Supplementary Materials

The following supporting information can be downloaded at: https://www.mdpi.com/article/10.3390/foods15213940/s1, Text S1: Conceptual comparison of single-mode and dual-mode electrochemical sensing configurations; Table S1: Conceptual comparison of single-mode and dual-mode electrochemical sensing configurations; Figure S1: Linear correlation plot of the DPV peak current increment (ΔI_p_) versus the EIS charge-transfer resistance increment (ΔR_ct_) across different OTA concentrations; Text S2: Optimization of Experimental Conditions Monitored via Voltammetry; Figure S2: DPV response curves for the optimization of key experimental parameters; Text S3: Electrochemical Kinetic Validation via Cyclic Voltammetry; Figure S3: Comparative cyclic voltammetry (CV) curves of the aptasensor at different modification stages; Text S4: Estimation of the Limits of Quantification.
