# Supplementary material for: A Dual-Mode Electrochemical Aptasensor for Ochratoxin A Detection in Red Wine Based on a Target-Induced Displacement Strategy
Source: Foods. 2026 Nov 3;15(21):3940. doi: 10.3390/foods15213940 (PMC13763166; doi:10.3390/foods15213940)
Supplement: Supplementary file 1 [file foods-15-03940-s001.zip › foods-4614696-supplementary.pdf]

Supporting Information

A Dual-Mode Electrochemical Aptasensor for Ochratoxin A Detection in Red Wine Based on a Target-Induced Displacement Strategy

Xueying Yu [1,2], Zhaoxian Hou [1,2], Yi Zhi [1,2], Shuo Zhang [1,2], Yuping Wen [2,*] and Zhijun Guo [1,2,*]

[1] Key Laboratory of Natural Medicines of the Changbai Mountain, Ministry of Education, Yanbian University, Yanji 133002, China
[2] College of Agriculture, Yanbian University, Yanji 133002, China
* Correspondence: 0000008711@ybu.edu.cn (Y.W.); guozhijunvip@163.com (Z.G.)

Text S1. Conceptual comparison of single-mode and dual-mode electrochemical sensing configurations.

Single-mode sensors (EIS-only or DPV-only) may be more susceptible to erroneous readouts in complex matrices because localized fouling, surface defects, or electronic drift can affect a single signal channel.

Table S1. Conceptual comparison of single-mode and dual-mode electrochemical sensing configurations.

Sensing Configuration	Primary Signal Readout	Potential Susceptibility to Matrix Interference	Verification Mechanism	Potential Risk of Erroneous Outcomes
EIS-only Mode	Interfacial charge-transfer resistance (R_{ct})	Potentially high (Vulnerable to non-specific adsorption or baseline drift)	None (Relies on a single-channel readout without independent verification)	Potentially high (Random artifacts may be misclassified as false positives)
DPV-only Mode	Peak oxidation current (I_p)	Potentially high (Vulnerable to ambient temperature or local fouling)	None (Relies on a single-channel readout without independent verification)	Potentially high (Random signal fluctuations may produce false negatives)

Proposed Dual-Mode	Synchronized readouts of both R_{ct} and I_p	Additional cross-check provided by cross-channel correlation	Cross-channel comparison based on the observed correlation between ΔRct and ΔI_p	Potentially reduced (Inconsistent single-channel responses can be identified through cross-channel comparison)

As indicated in Table S1, matrix-related perturbations may influence one electrochemical channel without complementary electrochemical readouts for comparison. In the proposed dual-mode system, the same target-induced interfacial change is monitored by both EIS and DPV, providing an additional cross-check through the observed correlation between ΔR_{ct} and ΔI_p.

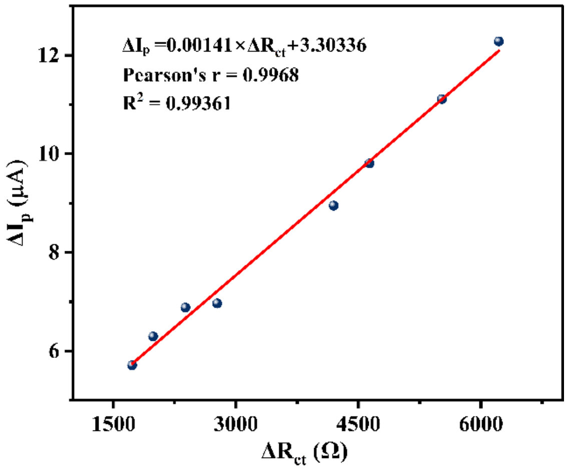

Figure S1. Linear correlation plot of the DPV peak current increment (ΔI_p) versus the EIS charge-transfer resistance increment (ΔR_{ct}) across different OTA concentrations.

Text S2. Optimization of Experimental Conditions Monitored via Voltammetry.

To establish an optimal microenvironment for interfacial molecular recognition, the critical parameters governing the biosensor fabrication and target-induced displacement (TID) kinetics were systematically optimized. While electrochemical impedance spectroscopy (EIS) served as the primary diagnostic tool, While EIS served as the primary diagnostic tool, the corresponding DPV responses were also examined

for consistency with the EIS-based optimization results.

As illustrated in Figure S2A, the DPV peak current (I_p) increased gradually as the cDNA concentration rose from 0.5 μM to 2.0 μM. Given the signal-on nature of this platform, this upward trend suggests that increasing the cDNA concentration within this range favors aptamer hybridization and enhances the subsequent target-induced displacement response. However, a further increase to 4.0 μM led to a noticeable decline in current, which may be attributed to excessive spatial crowding and electrostatic repulsion among densely packed cDNA strands, which may hinder subsequent aptamer hybridization.

Similarly, the DPV response reached a maximum at 120 min (Figure S2B), beyond which the current response plateaued or slightly decreased, possibly due to increased steric hindrance in a densely packed cDNA layer. Furthermore, the kinetics of the OTA-induced displacement reaction were monitored over 15–90 min (Figure S2C). The DPV peak current increased steadily and reached a stable response at 60 min. Consequently, a cDNA concentration of 2.0 μM, an immobilization time of 120 min, and an OTA incubation interval of 60 min were selected as the optimal conditions.

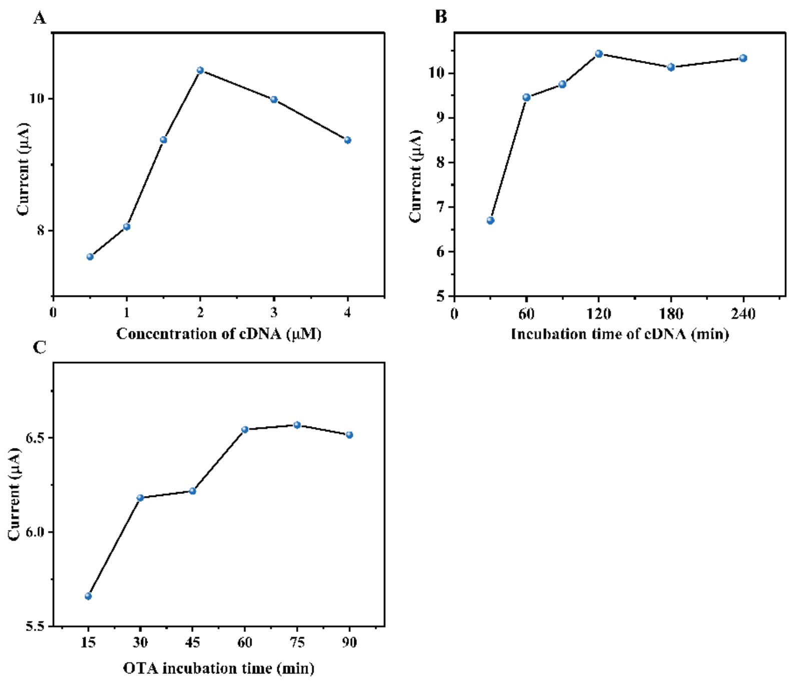

Figure S2. DPV response curves for the optimization of key experimental parameters. Effects of (A) cDNA immobilization concentration, (B) cDNA immobilization time, and (C) OTA incubation time on the DPV peak currents (I_p). All measurements were performed under the same experimental conditions as the EIS optimizations.

Text S3. Electrochemical Characterization by Cyclic Voltammetry

To further assess the interfacial electron-transfer kinetics during the stepwise assembly and target-induced displacement processes, cyclic voltammetry (CV) was performed in a 5.0 mM $[Fe(CN)_6]^{3-/4-}$ solution containing 0.4 M KNO_3 at a scan rate of 50 mV/s.

As depicted in Figure S3, the bare gold electrode exhibited a well-defined, reversible pair of redox peaks with a relatively small peak-to-peak potential separation (ΔE_p). Upon the successive immobilization of thiolated cDNA, passivation with MCH, and hybridization with the aptamer, the oxidation and reduction peak currents (I_p) decreased sequentially, accompanied by a progressive expansion of the ΔE_p value. This behavior is consistent with the formation of a dense, negatively charged dsDNA barrier layer that restricts the mass transport of the anionic probe toward the electrode.

Crucially, upon incubation with OTA, the target-induced displacement of the aptamer led to a distinct recovery of the redox peak currents and a corresponding narrowing of ΔE_p. This kinetic recovery indicates that the interface became more accessible to the redox probe, supporting the proposed target-induced displacement mechanism. Although CV and ΔE_p analyses provide complementary electrochemical evidence consistent with the proposed interfacial mechanism, they do not directly establish the chemical composition or topography of the electrode surface.

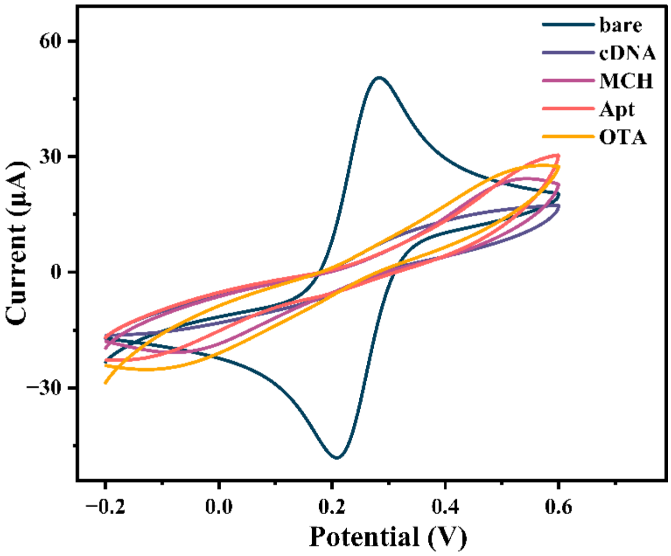

Figure S3. Comparative cyclic voltammetry (CV) curves of the aptasensor at different modification stages: bare, cDNA/Au, MCH/cDNA/Au, Apt/MCH/cDNA/Au, and OTA/Apt/MCH/cDNA/Au electrodes in 5.0 mM $[Fe(CN)_6]^{3-/4-}$ containing 0.4 M KNO_3. Scan rate: 50 mV/s.

Text S4. Estimation of the Limits of Quantification

The limits of quantification (LOQs) were estimated using a 10σ signal-threshold criterion based on blank-signal variability:

$$y_{th} = \begin{cases} \bar{y}_{blank} - 10\sigma, & EIS \\ \bar{y}_{blank} + 10\sigma, & DPV \end{cases}$$

$$C_{LOQ} = 10^{(y_{th}-a)/b}$$

where C_{LOQ} is the estimated OTA concentration in ng/mL, $\bar{y}_{blank}$ is the mean blank signal, σ is the standard deviation of the blank responses, and a and b are the intercept

and slope, respectively, of the calibration equation $y = a + b \times \log_{10}(C)$. The minus sign was used for EIS, whereas the plus sign was used for DPV, according to the direction of signal change with increasing OTA concentration.

The estimated LOQs were 0.0228 ng/mL for EIS and 0.1097 ng/mL for DPV. The EIS LOQ was below the lowest experimentally tested concentration (0.05 ng/mL) and should therefore be regarded as an extrapolated estimate, whereas the DPV LOQ fell within the investigated calibration range of 0.05–10 ng/mL. Quantitative accuracy and precision were not specifically validated at the estimated LOQ levels.
